# Impact of Early Surgery on Clinical Outcomes of Super‐Aged Patients With Hip Fractures: A Retrospective Propensity Score‐Matched Study With 2‐Year Follow‐Up

**DOI:** 10.1111/os.70267

**Published:** 2026-02-10

**Authors:** Tian Xie, Chen Rui, Wang Gao, Yucheng Gao, Chuwei Tian, Liu Shi, Wenbin Fan, Hui Chen, Yunfeng Rui

**Affiliations:** ^1^ Department of Orthopedics Zhongda Hospital Affiliated to Southeast University Nanjing China; ^2^ Orthopaedic Trauma Institute, Zhongda Hospital Affiliated to Southeast University Nanjing China; ^3^ Trauma Center, School of Medicine Zhongda Hospital Affiliated to Southeast University Nanjing China; ^4^ Multidisciplinary Team for Geriatric Hip Fracture Comprehensive Management Zhongda Hospital Affiliated to Southeast University Nanjing China

**Keywords:** early surgery, hip fracture, super‐aged

## Abstract

**Objective:**

The global aging population has led to a significant increase in hip fractures among elderly patients, posing substantial clinical challenges. While early surgical intervention is widely advocated, its impact on postoperative complications and mortality in super‐aged (≥ 80 years) hip fracture patients remains controversial. This study aimed to evaluate the association between early surgery and clinical outcomes in this population.

**Methods:**

We conducted a retrospective cohort study of patients aged ≥ 80 years who underwent hip fracture surgery at a single‐center orthopedic trauma center between January 2018 and November 2021. Participants were stratified into early surgery (≤ 48 h post‐admission) and non‐early surgery groups. Propensity score matching (PSM) was employed to control for confounding variables. Primary outcomes included 30‐day, 90‐day, 1‐year, and 2‐year mortality rates. Secondary outcomes encompassed perioperative transfusion rates, postoperative complications, hospital length of stay (LOS), and hospitalization costs.

**Results:**

After PSM, a total of 300 patients were included. Compared with the non‐early surgery group, the early surgery group had lower 1‐year (11.6% vs. 28.0%, *p* < 0.001) and 2‐year (36.0% vs. 50.7%, *p* = 0.010) postoperative mortality rates, a lower perioperative blood transfusion rate (32.7% vs. 53.3%, *p* < 0.001), lower incidences of postoperative pneumonia (15.3% vs. 29.3%, *p* = 0.004) and delirium (14.0% vs. 36.0%, *p* < 0.001), a shorter length of stay [8.6 days (7.5, 11.2) vs. 11.6 days (9.7, 14.9), *p* < 0.001], and lower hospitalization expenses [54,336 ¥ (48,965, 64,532) vs. 61,616 ¥ (50,758, 74,484), *p* = 0.001]. The serum albumin level at discharge in the early surgery group was higher (33.4 (31.6, 35.4) vs. 32.6 (30.7, 34.9), *p* = 0.039). Kaplan–Meier survival curve analysis showed that the all‐cause mortality rate in the non‐early surgery group increased (Log Rank *p* = 0.0066). Multivariate Cox analysis showed that age, BMI, admission hemoglobin, and non‐early surgery were risk factors for 2‐year mortality.

**Conclusion:**

Early surgical intervention for hip fractures in super‐aged patients is associated with improved survival, reduced complications, and better resource utilization. These findings support the implementation of protocols to minimize preoperative delays in this vulnerable population.

## Graphical abstract

Early surgery (< 48 h) in super‐aged hip fracture patients reduces 1–2 years mortality, complications, hospital stay, and costs, supporting timely intervention protocols.

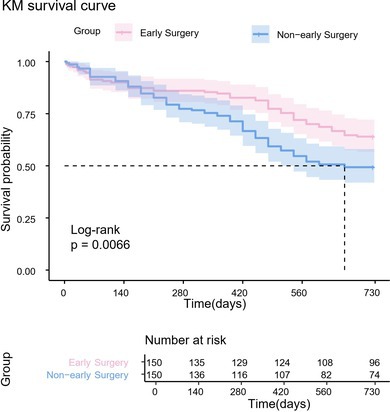

## 1 Background

Hip fractures, primarily consisting of femoral neck, intertrochanteric, and subtrochanteric fractures [1], represent a significant healthcare burden in aging populations. With the ongoing demographic shift toward older age groups worldwide, the incidence of hip fractures has risen dramatically [2]. Current projections estimate that by 2050, over 6 million individuals globally will require hospitalization annually for hip fractures, creating substantial socioeconomic impacts [3, 4].

The management of elderly hip fracture patients presents two main treatment approaches: conservative management and surgical intervention [5]. Current evidence demonstrates that conservative treatment carries considerable risks. A meta‐analysis revealed that over one‐third of elderly hip fracture patients managed conservatively developed in‐hospital complications, with mortality rates reaching 36%, 46%, and 60% at 1 month, 6 months, and 1 year, respectively [6]. Furthermore, conservative treatment has been associated with a 2.8‐fold increased risk of 30‐day mortality compared to surgical intervention [7]. As a result, surgical treatment—including total hip arthroplasty (THA), hip hemiarthroplasty (HHA), proximal femoral nail antirotation (PFNA), and dynamic hip screw (DHS)—remains the preferred option for medically fit patients [8, 9, 10].

The epidemiology of hip fractures shows a clear age‐dependent pattern. Among elderly populations, those aged 80 years and older (the “super‐aged” group) account for the majority of hip fracture cases [11, 12, 13]. While some studies report significantly higher mortality in this age group [14, 15], others suggest a more complex, non‐linear relationship between age and outcomes [16]. Interestingly, patients aged 80–90 years typically present with greater comorbidity burdens and worse outcomes than younger elderly patients (65–80 years), while those over 90 often show fewer comorbidities (such as diabetes) and better prognoses—a phenomenon potentially explained by survivor selection effects [16].

The optimal timing of surgical intervention for patients with hip fractures remains controversial. A previous study using the Danish Fracture Database found that for patients with proximal femoral fractures, surgical delays exceeding 12 h increased the risk of 30‐day mortality, and delays beyond 24 h elevated the risk of 90‐day mortality [17]. This suggests that early surgery, particularly within 48 h of admission, has seemingly become the mainstream approach [18, 19, 20]. However, other literature has pointed out that since most hip fracture patients are elderly with multiple comorbidities, preoperative optimization is crucial—and surgical delays resulting from such optimization do not increase postoperative complications or mortality [21, 22, 23]. A recent retrospective multicenter cohort study involving 7414 elderly patients with intertrochanteric femoral fractures demonstrated that surgical intervention delayed by 48 h did not lead to higher mortality or poorer functional outcomes [24]. While numerous studies have explored surgical intervention within 36 h or even 24 h post‐injury [25, 26], these works primarily focus on broader elderly populations (≥ 65 years) and lack dedicated analysis of the super‐aged subgroup (≥ 80 years). For the super‐aged population, the selection of surgical timing is even more complex due to the unique relationship between their age and prognosis [16]. Furthermore, most existing literature emphasizes short‐term outcomes (e.g., 30‐day mortality) rather than postoperative complications and long‐term survival indicators. These research gaps highlight the necessity of conducting targeted studies to evaluate the efficacy and safety of early surgery in super‐aged hip fracture patients.

Given the controversies among the aforementioned studies and the current near‐absence of long‐term follow‐up studies focusing on the special subgroup of super‐aged patients with hip fractures, this study aims to: (i) systematically evaluate the association between early surgical intervention (≤ 48 h post‐admission) and key clinical outcomes (including long‐term mortality, postoperative complications, hospital resource utilization, and nutritional recovery) in super‐aged patients (≥ 80 years) with hip fractures; (ii) identify independent risk factors for 2‐year mortality in this vulnerable population through multivariate analysis; and (iii) provide evidence‐based support for optimizing clinical decision‐making regarding surgical timing in super‐aged patients with hip fractures.

## 2 Patients and Methods

### 2.1 Study Design, Setting, and Population

A retrospective study was performed at a single‐center orthopedic trauma center from January 2018 to November 2021. This study has been approved by the Independent Ethics Committee for Clinical Research (IEC). Additionally, the Institutional Review Board (IRB) granted a waiver of informed consent on the basis that this study would not adversely affect the rights or welfare of the participants (2022ZDSYLL183‐P01). All data were anonymized prior to analysis to protect patient privacy.

Inclusion criteria: (1) Age ≥ 80 years; (2) Fracture type of femoral neck fracture or intertrochanteric fracture; (3) Fresh fracture (fracture time ≤ 3 weeks); (4) Received surgical treatment (all patients with femoral neck fracture received THA or HHA, and all patients with intertrochanteric fracture received internal fixation).

Exclusion criteria: (1) Surgical procedure other than arthroplasty or intramedullary nail fixation; (2) Patients with bilateral hip fracture; (3) High‐energy injuries; (4) Open fracture; (5) Pathological fracture; (6) Patients with loss of visit.

**FIGURE 1:**
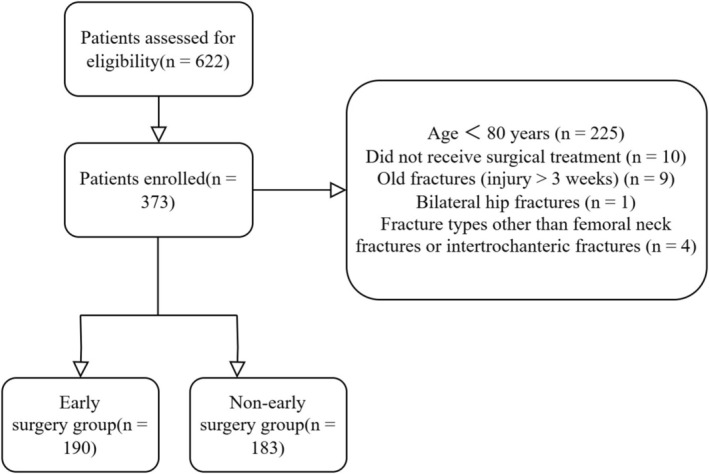
The flow diagram of this study.

After screening according to the inclusion and exclusion criteria, a total of 373 patients were included in this study, including 109 males and 264 females, with a mean age of (86.0 ± 4.0) years. According to previous literature, early surgery was categorized based on the waiting time from admission to surgery [17, 18, 19]. Specifically, patients who underwent surgery within 48 h of admission were divided into the early surgery group (*n* = 190), and the remaining patients were included in the non‐early surgery group (*n* = 183). The flow diagram of the included patients in this research is shown in Figure 1. This is an observational study.

### 2.2 Perioperative Treatment and Surgical Procedure

Our center offers 24/7 support, including weekends and national holidays, with a minimum of two orthopedic surgeons available on the ward every day. We have a Multidisciplinary Team (MDT) comprising 18 core departments, including Orthopedic Trauma, Intensive Care Unit (ICU), Anesthesia, and others, which are actively involved throughout the entire process of assessment and treatment for elderly hip fracture patients. This includes the emergency, pre‐operative, intra‐operative, post‐operative, and discharge follow‐up phases [27]. Furthermore, our MDT has established an ICU Fast‐Track specifically for elderly hip fracture patients, enabling those in poor general condition to be promptly transferred to the ICU for intensive care.

During the perioperative period, all patients undergo comprehensive assessments along with standardized diagnosis and treatment protocols. Patients undergoing surgical intervention are treated by the same surgical team. For femoral neck fractures, surgeries such as Total Hip Arthroplasty (THA), Hip Hemiarthroplasty (HHA), or Dynamic Hip Screw (DHS) are performed. For intertrochanteric femoral fractures, surgeries like Proximal Femoral Nail Anti‐Rotation (PFNA) are carried out. Post‐operatively, patients are encouraged to engage in partial or full weight‐bearing activities as soon as possible, with assistance provided when necessary.

### 2.3 Data Collection

The data were retrospectively collected from the electronic patient records at the institution by two orthopedic surgeons. Demographic data included sex, age, body mass index (BMI), and general health status according to the American Society of Anaesthesiologists (ASA) classification [28], smoking history, and comorbidities (consisting of hypertension, diabetes, coronary heart disease, cerebral infarction, deep vein thrombosis, renal insufficiency, osteoporosis). The diagnosis of osteoporosis was uniformly based on the results of dual‐energy X‐ray absorptiometry (DXA) scans [29]. The injury‐related data included fracture type (femoral neck fracture or intertrochanteric fracture), time from injury to admission, and laboratory examinations at admission. The surgery‐related data included type of surgery, type of anesthesia, intraoperative blood loss, and duration of surgery (THA, HHA, multiple screws and intramedullary nail fixation). The in‐hospital data included total hospital costs (THC), length of stay (LOS), short‐term postoperative complications, and laboratory examinations after surgery. A dedicated follow‐up team is available in our department, and relevant follow‐up instructions are provided to patients and their family members prior to the patients' discharge. The follow‐up started after surgery, and the endpoint events were defined as all‐cause mortality or end of study, whichever was earlier. The last follow‐up of this study was completed in November 2023, and all 300 super‐aged patients successfully completed a 24‐month follow‐up.

### 2.4 Outcome Measures

The main outcome indicators of this study include the mortality rates at 30 days, 90 days, 1 year, and 2 years postoperatively. The secondary outcome indicators include the perioperative blood transfusion rate, the incidence rates of postoperative complications (postoperative pneumonia, postoperative delirium), the total length of hospital stay, the length of postoperative hospital stay, the hospitalization expenses, and the levels of hemoglobin (HB), albumin (ALB), and creatinine at the time of discharge.

All patients were followed up from discharge until the date of death or the end of the study. Time, cause of death, 30 days all‐cause mortality, 90 days all‐cause mortality, 1 year all‐cause mortality, 2 years all‐cause mortality were recorded. The follow‐up results were based on the outpatient clinical system and telephone contact with patients and their family members.

### 2.5 Statistical Analysis

We evaluated the distributions of all continuous variables for normality by using the Shapiro–Wilk test. Data satisfying normalcy are presented as the mean and standard deviation (SD). Nonnormally distributed variables are presented as the median (IQR). Categorical variables are shown as counts (percentages). The overall data were analyzed by Student's *t*‐test or the Mann–Whitney U test for continuous variables and the chi‐square test for categorical variables, as appropriate. Univariate and multivariate analyses were used to further test for the independent influencing factors of prolonged time to surgery. Differential analysis was conducted between the two groups using the original data. For datasets with imbalanced baselines, propensity score matching (PSM) was performed at a 1:1 ratio with a matching tolerance of 0.02. Kaplan–Meier survival analysis was used to compare the survival time between the two groups. Multivariate Cox regression analysis was performed to identify factors associated with mortality in patients and adjust for residual confounding factors; the candidate variables for this analysis were derived from previously published literature focusing on risk factors for mortality in hip fracture [30, 31, 32]. All statistical analyses were performed using SPSS statistical software (version 26.0, SPSS Inc., Chicago, USA). A two‐sided *p*‐value < 0.05 was considered significant.

## 3 Results

### 3.1 Population and Patient Characteristics

A total of 373 patients were included in the final analysis. The baseline data are presented in Table 1. Both groups of patients completed a follow‐up period of 2 years. Among them, there were significant statistical differences in diabetes, renal insufficiency, operation duration, platelet count at admission, and FIB‐C (*p* < 0.05). There were no significant statistical differences in other baseline data (*p* > 0.05). PSM was performed based on the characteristics with differences between the two groups of patients (diabetes, renal insufficiency, operation duration, platelet count, and FIB‐C) at a matching ratio of 1:1 and a matching tolerance of 0.02. A total of 300 patients were successfully matched, with 150 cases in the early surgery group and 150 cases in the non‐early surgery group. Further statistical analysis showed that there were no statistical differences in the baseline data between the two groups of patients after matching (*p* > 0.05) (Table 2).

**TABLE 1:** Characteristics of patients before and after PSM.

Group	Before PSM			After PSM		
	Early surgery group (*n* = 190)	Non‐early surgery group (*n* = 183)	*p*	Early surgery group (*n* = 150)	Non‐early surgery group (*n* = 150)	*p*
Age (years)	86 (83, 89)	85 (83, 89)	0.360	86 (83, 90)	85 (83, 89)	0.288
Gender						
Male	53 (27.9)	56 (30.6)	0.566	53 (35.3)	44 (29.3)	0.267
Female	137 (72.1)	127 (62.4)		94 (64.7)	106 (70.7)	
BMI (kg/m^2^)	21.7 (19.6, 25.0)	22.5 (20.0, 24.6)	0.435	22.4 ± 4.3	22.2 ± 3.4	0.692
ASA grade						
I/II	80 (42.1)	74 (40.4)	0.744	62 (41.3)	72 (48.0)	0.907
III/IV	110 (57.9)	109 (59.6)		88 (58.7)	78 (52.0)	
Comorbidities						
Hypertension	115 (60.5)	109 (59.6)	0.849	91 (60.7)	88 (58.7)	0.724
Diabetes	36 (18.9)	51 (27.9)	0.042*	34 (22.7)	34 (22.7)	1.000
Coronary heart disease	42 (22.1)	35 (19.1)	0.477	33 (22.0)	29 (19.3)	0.568
Cerebral infarction	69 (36.3)	72 (39.3)	0.546	53 (35.3)	60 (40.0)	0.404
Deep vein thrombosis	41 (21.6)	36 (19.7)	0.649	34 (22.7)	32 (21.3)	0.780
Renal insufficiency	2 (1.1)	9 (4.9)	0.027*	2 (1.3)	2 (1.3)	1.000
Osteoporosis	120 (63.2)	107 (58.5)	0.354	94 (62.7)	87 (58.0)	0.409
Fracture type						
Femoral neck fracture	74 (38.9)	85 (46.4)	0.143	62 (41.3)	72 (48.0)	0.246
Intertrochanteric fracture	116 (61.1)	98 (53.6)	0.491	88 (58.7)	78 (52.0)	0.646
Time from injury to hospital admission (h)	9.5 (5.0, 24.0)	9.0 (5.0, 24.0)	0.776	10 (5, 24)	8 (4, 24)	0.156
Anesthesia type						
General anesthesia	173 (91.1)	164 (89.6)	0.639	139 (92.7)	133 (88.7)	0.234
Regional anesthesia	17 (8.9)	19 (10.4)		11 (7.3)	17 (11.3)	
Surgical procedure						
THA	11 (5.8)	14 (7.7)	0.535	11 (7.3)	13 (8.7)	0.861
HHA	63 (33.2)	67 (36.6)		52 (34.7)	54 (36.0)	
Intramedullary nail fixation	116 (61.1)	102 (55.7)		87 (58.0)	83 (55.3)	
Surgical duration (min)	90 (79.8, 105.0)	95 (79.8, 120.0)	0.014*	90 (80.0, 109.8)	90 (79.8, 109.2)	0.923
Intraoperative blood loss (mL)	110 (100, 200)	100 (100, 200)	0.735	150 (100, 200)	100 (100, 200)	0.202
Results of first laboratory tests upon admission						
Red blood cell count (× 10^12^/L)	3.61 (3.25, 4.09)	3.73 (3.29, 4.06)	0.615	3.68 (3.26, 4.12)	3.73 (3.24, 4.09)	0.937
Hemoglobin (g/L)	114 (102, 128)	114 (103, 127)	0.843	114 (103, 128)	115 (105, 127)	0.786
Hematocrit (%)	33.4 (29.6, 37.9)	33.3 (29.8, 37.3)	0.729	33.8 (30.0, 37.9)	33.4 (29.5, 37.3)	0.530
White blood cell count (× 10^9^/L)	9.01 (6.88, 11.25)	8.63 (7.25, 11.04)	0.858	9.17 (6.93, 11.25)	8.53 (7.20, 11.14)	0.591
Neutrophil ratio (%)	83.0 (78.2, 88.6)	83.5 (76.6, 88.0)	0.666	82.8 (78.1, 87.6)	84.6 (77.0, 89.1)	0.599
Platelet count (× 10^9^/L)	170 (140, 208)	178 (153, 230)	0.014*	179.0 (140.3, 216.3)	177.0 (151.0, 225.3)	0.466
Total protein (g/L)	66.8 ± 9.6	66.4 ± 9.4	0.527	67.3 (59.1, 73.6)	68.6 (61.8, 73.7)	0.491
Albumin (g/L)	37.8 (34.6, 41.2)	38.0 (35.3, 40.4)	0.790	37.6 (34.1, 41.7)	38.1 (35.3, 40.8)	0.460
Lactate dehydrogenase (U/L)	258.5 (218.5, 316.3)	267.0 (218.0, 324.0)	0.440	256.5 (218.3, 316.3)	267.5 (217.8, 324.3)	0.440
Urea (mmol/L)	6.7 (5.4, 8.5)	7.1 (5.5, 9.2)	0.304	6.9 (5.5, 8.8)	6.9 (5.4, 8.9)	0.943
Creatinine (μmol/L)	67 (54, 84)	70 (56, 90)	0.299	68 (56, 84)	71 (56, 90)	0.544
Alanine aminotransferase (U/L)	21 (14, 27)	22 (15, 28)	0.224	20 (14, 26)	22 (15, 28)	0.265
Aspartate aminotransferase (U/L)	27 (23, 33)	27 (22, 34)	0.987	27 (23, 33)	27 (22, 34)	0.847
Sodium ion (mmol/L)	138.1 (135.2, 140.7)	137.6 (135.6, 139.9)	0.378	138.1 (135.1, 140.9)	137.9 (135.6, 140.0)	0.634
Potassium ion (mmol/L)	3.72 (3.42, 4.07)	3.82 (3.49, 4.12)	0.365	3.72 (3.40, 4.09)	3.82 (3.48, 4.10)	0.521
Calcium ion (mmol/L)	2.18 (1.98, 2.28)	2.20 (2.04, 2.27)	0.265	2.17 (1.95, 2.28)	2.21 (2.07, 2.28)	0.110
Chloride ion (mmol/L)	105.0 (102.0, 107.3)	103.9 (101.3, 106.9)	0.093	105.1 (102.1, 107.6)	104.0 (101.5, 106.9)	0.126
PT (s)	11.7 (11.3, 12.5)	11.9 (11.3, 12.4)	0.173	11.7 (11.3, 12.4)	11.9 (11.4, 12.4)	0.142
APTT (s)	30.0 (27.5, 32.4)	29.7 (27.5, 32.6)	0.637	30.0 (27.4, 32.2)	29.7 (27.5, 32.7)	0.986
INR	1.09 (1.05, 1.18)	1.11 (1.06, 1.18)	0.185	1.10 (1.05, 1.18)	1.11 (1.07, 1.18)	0.205
FIB‐C (g/L)	3.6 (3.1, 4.2)	3.8 (3.2, 4.4)	0.030*	3.6 (3.1, 4.4)	3.8 (3.2, 4.4)	0.198
FDP (mg/L)	31.8 (10.4, 76.9)	31.3 (9.9, 80.1)	0.644	30.8 (10.1, 74.0)	35.4 (11.8, 93.1)	0.513
ATIII (%)	85 (78, 96)	88 (77, 99)	0.369	85 (78, 96)	88 (79, 98)	0.240
D‐dimer (μg/L)	5892 (1713, 12,743)	5280 (1405, 9864)	0.158	6209 (1639, 12,647)	5814 (1767, 11,402)	0.479

*Note*: A *p*‐value < 0.05.

Abbreviation: SD, standard deviation.

*Indicates statistical significance.

**TABLE 2:** Patient outcome analyses after propensity score matching.

Variables	Early surgery group (*n* = 150)	Non‐early surgery group (*n* = 150)	*p*
Primary outcome indicators			
30‐day mortality	6 (4.0)	3 (2.0)	0.310
90‐day mortality	14 (9.3)	11 (7.3)	0.531
1‐year mortality	17 (11.6)	42 (28.0)	< 0.001*
2‐year mortality	54 (36.0)	76 (50.7)	0.010*
Secondary outcome indicators			
Perioperative transfusion rate	49 (32.7)	80 (53.3)	< 0.001*
Postoperative pneumonia	23 (15.3)	44 (29.3)	0.004*
Postoperative delirium	21 (14.0)	54 (36.0)	< 0.001*
LOS (d)	8.6 (7.5, 11.2)	11.6 (9.7, 14.9)	< 0.001*
Postoperative LOS (d)	7.0 (6.0, 9.8)	7.7 (6.4, 10.0)	0.187
THC (¥)	54,336 (48,965, 64,532)	61,616 (50,758, 74,484)	0.001*
Hemoglobin at discharge (g/L)	97.0 (88.0, 108.0)	96.0 (87.8, 106.0)	0.603
Albumin at discharge (g/L)	33.4 (31.6, 35.4)	32.6 (30.7, 34.9)	0.039*
Creatinine at discharge (μmol/L)	69 (59, 92)	71 (57, 89)	0.681

*Note*: A *p*‐value < 0.05.

Abbreviation: LOS, length of hospital stay.

*Indicates statistical significance.

### 3.2 Outcome Indicators for the Early Surgery Group and the Non‐Early Surgery Group

After propensity score‐based matching, compared with the non‐early surgery group, the early surgery group had lower rates of 1 year postoperative mortality (11.6% vs. 28.0%), 2 years postoperative mortality (36.0% vs. 50.7%), perioperative blood transfusion rate (32.7% vs. 53.3%), incidence of postoperative pneumonia (15.3% vs. 29.3%), incidence of postoperative delirium (14.0% vs. 36.0%), LOS [8.6 days (7.5, 11.2) vs. 11.6 days (9.7, 14.9)], and THC [54,336 ¥ (48,965, 64,532) vs. 61,616 ¥ (50,758, 74,484)]. The albumin level at discharge in the early surgery group was higher than that in the non‐early surgery group [33.4 g/L (31.6, 35.4) vs. 32.6 g/L (30.7, 34.9)]. All these differences were statistically significant (*p* < 0.05). There were no significant statistical differences between the two groups of patients in 30 days postoperative mortality, 90 days postoperative mortality, length of postoperative hospital stay, hemoglobin level at discharge, and creatinine level (Table 2).

### 3.3 Survival Curves

A follow‐up of 24 months was conducted for 300 super‐aged patients. Among them, 59 patients died within 12 months, and 130 patients died within 24 months. The two‐year mortality rate was 43.3%, and the annual mortality rate due to fractures was 19.7%. The most common causes of death were cardiovascular events and pneumonia.

**FIGURE 2:**
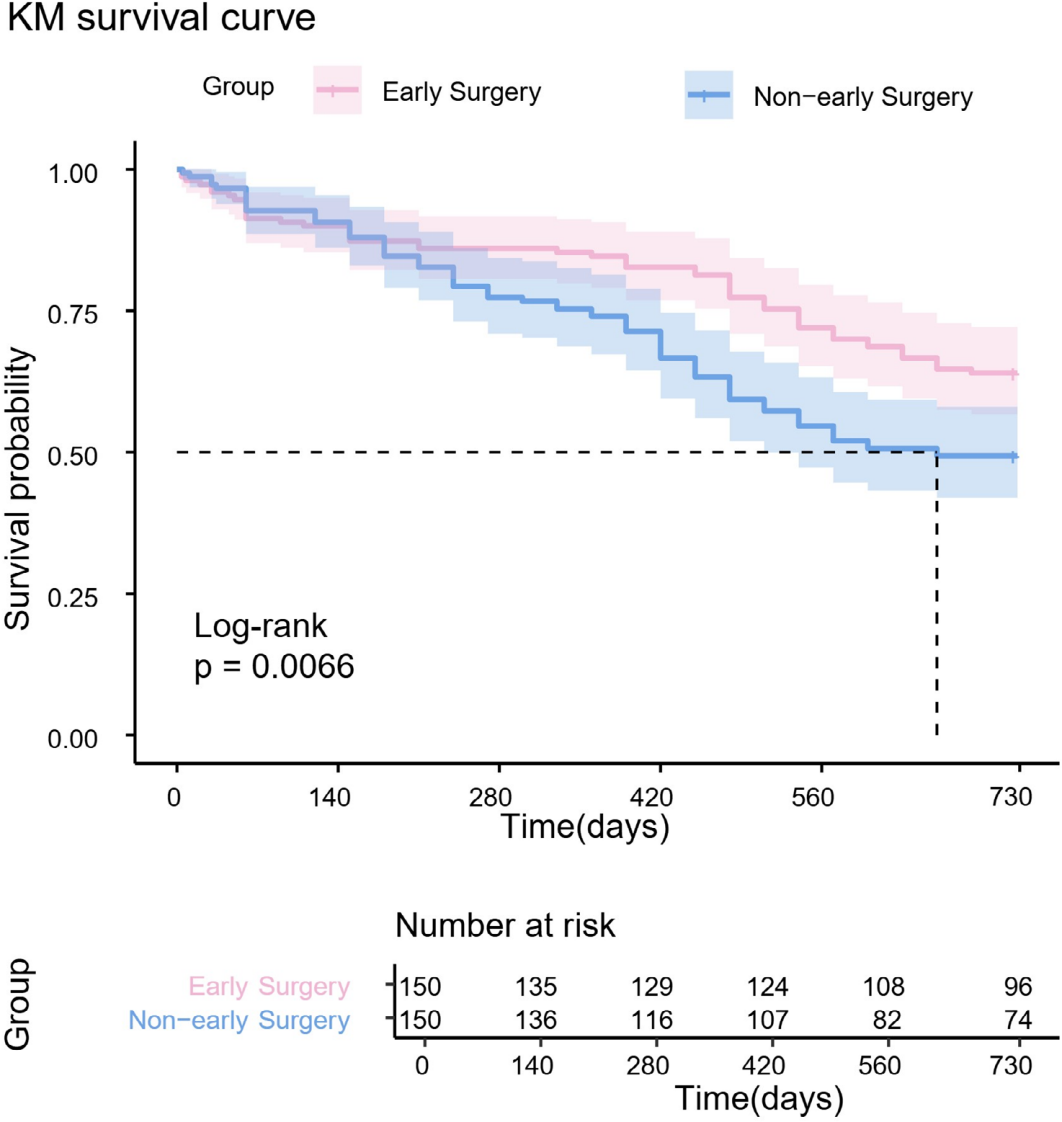
Kaplan–Meier survival curve.

The Kaplan–Meier survival curve analysis showed that the all‐cause mortality rate in the non‐early surgery group of super‐aged elderly patients with hip fractures increased (*p* = 0.0066, Figure 2).

### 3.4 Multivariate Cox Regression Analysis

A multivariate Cox proportional hazards model was used to estimate the effect of each factor on cumulative survival rate. The factors evaluated in this model included age, gender, BMI, ASA classification, hypertension, diabetes, coronary heart disease, cerebral infarction, deep vein thrombosis, renal insufficiency, osteoporosis, admission hemoglobin, and non‐early surgery.

Among super‐aged patients with hip fracture, during a follow‐up period of 24 months, age (*p* < 0.001), BMI (*p* = 0.038), admission hemoglobin (*p* = 0.026), and non‐early surgery (*p* = 0.002) were associated with all‐cause mortality (Table 3).

**TABLE 3:** Multivariate Cox proportional analysis of risk factors for 2‐year mortality in hip fracture patients.

Variable	Hazard ratio	95% CI	*p*
Age	1.113	1.066–1.163	< 0.001*
BMI	0.951	0.906–0.997	0.038*
Admission hemoglobin	0.990	0.981–0.999	0.026*
Non‐early surgery	1.754	1.224–2.514	0.002*

*Note*: A *p*‐value < 0.05.

*Indicates statistical significance.

## 4 Discussion

Unlike previous studies investigating the surgical timing for hip fractures, the present study centered on the specific population of super‐aged patients with hip fractures, conducting long‐term follow‐up and performing a comprehensive assessment of their postoperative outcomes. Our study found that early surgery in super‐aged patients was associated with reduced 1‐year and 2‐year mortality rates, lower perioperative transfusion requirements, decreased incidence of postoperative complications (particularly pneumonia and delirium), shorter hospital stays, and reduced hospitalization costs. Multivariate Cox regression analysis demonstrated that 2‐year mortality in super‐aged patients with hip fractures was associated with non‐early surgery, age, BMI, and admission hemoglobin.

### 4.1 Early Surgery and Its Association With Key Clinical Outcomes in Super‐Aged Hip Fracture Patients

Hip fractures represent a major healthcare challenge in elderly populations, associated with high rates of disability, mortality, and substantial medical costs [4, 33]. These injuries severely impact patients' quality of life while placing significant burdens on families and society. As populations continue to age, the incidence of hip fractures among the “older elderly” has risen markedly. Epidemiological studies indicate that 17% of men over 80 years will experience hip fractures [34], with individuals in this age group facing significantly elevated fracture risks regardless of gender—hip fractures rank among the top three most common fracture types in this population [35].

Current treatment guidelines recommend surgical intervention within 48 h of admission (early surgery) as the preferred approach, aiming to reduce postoperative complications, lower mortality rates, and improve overall prognosis [36]. However, some studies caution against indiscriminate early surgery for patients with poor baseline conditions or multiple comorbidities, emphasizing the need for thorough preoperative optimization to enhance surgical tolerance [37]. These considerations highlight the critical importance of carefully evaluating surgical timing in super‐aged hip fracture patients.

The relationship between surgical timing and outcomes remains controversial in existing literature. Some studies report a linear association between preoperative delay and increased mortality, with estimates suggesting a 5% rise in 1‐year mortality for every 10‐h delay [38]. Colais et al. [39] observed lower 1‐year mortality in patients operated within 48 h, while others have noted that delays in medical transfer significantly prolong preoperative waiting times and worsen outcomes [40]. However, conflicting evidence suggests that when delays result from necessary medical optimization for patients with active comorbidities, exceeding 48 h may not adversely affect outcomes [21, 41]. Our findings differ from these latter studies, possibly because all patients in our cohort received perioperative management through a multidisciplinary team approach, enabling rapid comprehensive optimization that may have mitigated negative impacts of medically necessary delays.

Regarding short‐term outcomes, while some studies demonstrate improved 30‐day survival with early surgery [19, 42, 43], we found no significant impact of surgical timing on short‐term mortality. This discrepancy may reflect our focus on an exceptionally elderly population (≥ 80 years) with greater comorbidity burdens and shorter life expectancies, potentially attenuating measurable effects of early intervention on short‐term outcomes.

Postoperative complications—particularly pneumonia and delirium—remain prevalent and clinically significant in this population [44, 45]. Our results align with previous findings that early surgery reduces pneumonia incidence [18] and that delays exceeding 48 h increase delirium risk [46]. After propensity score matching, we confirmed that delayed surgery significantly elevates the risk of both complications in super‐aged patients, reinforcing the argument for timely intervention in this vulnerable group.

### 4.2 2‐Year Mortality Risk Factors in Super‐Aged Hip Fracture Patients

Results of the multivariate Cox regression analysis revealed that the risk factors for all‐cause mortality in super‐aged patients with hip fractures were non‐early surgery, age, BMI, and admission hemoglobin. With increasing age, physical function gradually declines, often accompanied by osteoporosis and sarcopenia [47, 48]; thus, age is widely recognized as a high‐risk factor for mortality in patients with hip fractures [30, 31]. Our results indicated that a higher BMI exerted a protective effect against long‐term mortality in super‐aged hip fracture patients, which was in line with the outcomes of a previous meta‐analysis [49]. A plausible explanation is that after experiencing the dual trauma of fracture and surgery, hip fracture patients develop a series of inflammatory responses that persist for a certain period [50, 51]. Patients with higher BMI typically have greater stress tolerance due to abundant fat reserves serving as an energy source [52], leading to relatively better long‐term prognosis. Meanwhile, admission hemoglobin was also identified as a risk factor for mortality, which was consistent with prior research [32, 53]. Patients with anemia often have poor nutritional status, and anemia may even impair the postoperative mobility of patients with hip fractures. Notably, early postoperative ambulation and rehabilitation are crucial for the prognosis of these patients [54]. In contrast to previous studies, male gender was not a risk factor in our analysis. This discrepancy might be attributed to differences in gender distribution across datasets or the insufficient sample size in this study, which may have prevented the detection of potential gender‐related differences. After adjusting for residual confounders, the risk of 2‐year all‐cause mortality in the non‐early surgery group remained 1.754 times higher than that in the early surgery group. This finding suggests that orthopedic surgeons should prioritize performing surgery within 48 h for super‐aged hip fracture patients to reduce the incidence of postoperative complications and improve long‐term patient prognosis.

### 4.3 Evidence‐Based Implications for Surgical Timing Decision‐Making

Consistent with most previous studies and guidelines, early surgery in this study was defined as a time interval of less than 48 h from admission to surgery (in‐hospital waiting time) [17, 19, 33]. For this time window, control by physicians is high; even for patients with multiple comorbidities, the preoperative waiting time can be shortened through preoperative multidisciplinary collaborative management. However, in recent years, several studies have proposed that the preoperative waiting time should be calculated from the time of injury to surgery. A study by Zhang et al. revealed an association between time from injury to admission and 1‐year mortality in elderly patients with hip fractures, with mortality rates being significantly higher when this interval exceeded 9 h [55]. Jiang et al. conducted a 5‐year follow‐up study and found that pre‐hospital delay (> 48 h) was associated with an increased risk of postoperative complications and higher 5‐year mortality in elderly patients with hip fractures [56]. These findings indicate that pre‐hospital waiting time is also a factor that cannot be ignored. Since this study focused more on the in‐hospital management of super‐aged patients with hip fractures, the impact of pre‐hospital waiting time was not analyzed. Future studies should place greater emphasis on the overall process management of hip fracture patients from injury to surgery, including enhancing awareness of the severity of hip fractures among primary healthcare institutions and patients, optimizing inter‐hospital transfer and in‐hospital diagnosis and treatment processes, reducing surgical waiting time, and improving the overall prognosis of elderly patients with hip fractures.

### 4.4 Limitations and Strengths

This study provides valuable insights into the management of super‐aged hip fracture patients through its focused examination of this high‐risk population and extended 2‐year follow‐up period. The demonstrated benefits of early surgery across multiple outcome measures support current guideline recommendations. However, this study also has certain limitations. First, as a retrospective study with a limited sample size, its results may be affected by recall bias and selection bias. Although propensity score matching helps balance the baseline characteristics of the two groups, this process may also lead to the loss of some data. Meanwhile, due to the lack of data on pre‐fracture functional status (such as the Barthel Index), cognitive status, and frailty indices, the aforementioned variables that may affect the prognosis of elderly patients with hip fractures were not included in the analysis, and this may result in the overestimation or underestimation of the conclusions. Finally, given the single‐center design of this study and the fact that our hospital has a well‐established MDT to support the comprehensive management of elderly patients with hip fractures, the generalizability of the study results may be restricted. These limitations suggest that future well‐designed, multicenter, prospective cohort studies with large sample sizes are needed to further validate the conclusions of this study.

## 5 Conclusion

In conclusion, our comprehensive analysis demonstrates that early surgical intervention (within 48 h after admission) in super‐aged hip fracture patients provides multiple clinical advantages, including reduced 1‐year and 2‐year mortality, fewer postoperative delirium and pneumonia, decreased transfusion needs, shorter LOS, lower hospitalization cost, and better nutritional recovery at discharge. These findings underscore the critical importance of implementing optimized clinical pathways to minimize preoperative delays while ensuring adequate medical preparation for this high‐risk population.

## Author Contributions

All authors had full access to the data in the study and take responsibility for the integrity of the data and the accuracy of the data analysis. Conceptualization: Tian Xie and Chen Rui. Methodology: Wang Gao and Yucheng Gao. Investigation: Tian Xie, Chen Rui, Wang Gao, and Yucheng Gao. Formal analysis: Chuwei Tian and Liu Shi. Resources: Liu Shi and Wenbin Fan. Writing – original draft: Tian Xie, Chen Rui, and Wang Gao. Writing – review and editing: Tian Xie, Chen Rui, and Yucheng Gao. Visualization: Hui Chen. Supervision: Yunfeng Rui. Funding acquisition: Yunfeng Rui.

## Funding

This work was supported by the Winfast Charity Foundation (YL20220525).

## Conflicts of Interest

The authors declare no conflicts of interest.

## Supporting information


**Table S1:** Multivariate Cox proportional analysis of risk factors for 2‐year mortality in hip fracture patients.

## Data Availability

The data that support the findings of this study are available on request from the corresponding author. The data are not publicly available due to privacy or ethical restrictions.
