# Supplementary material for: Impact of Early Surgery on Clinical Outcomes of Super‐Aged Patients With Hip Fractures: A Retrospective Propensity Score‐Matched Study With 2‐Year Follow‐Up
Source: Orthop Surg. 2026 Feb 10;18(3):533–43. doi: 10.1111/os.70267 (PMC12967684; doi:10.1111/os.70267)
Supplement: Supplementary file 1 — Table S1: Multivariate Cox proportional analysis of risk factors for 2‐year mortality in hip fracture patients. [file OS-18-533-s001.docx]

Table S1 Multivariate Cox proportional analysis of risk factors for 2-year mortality in hip fracture patients

| **Variable** | **Hazard ratio** | **95% CI** | **P Value** |
| --- | --- | --- | --- |
| Age | 1.113 | 1.066-1.163 | <0.001* |
| Gender | 1.153 | 0.793-1.676 | 0.457 |
| BMI | 0.951 | 0.906-0.997 | 0.038* |
| ASA grade | 1.210 | 0.804-1.821 | 0.360 |
| Hypertension | 1.009 | 0.689-1.477 | 0.964 |
| Diabetes | 0.909 | 0.585-1.412 | 0.671 |
| Coronary Heart Disease | 0.990 | 0.619-1.583 | 0.967 |
| Cerebral Infarction | 1.412 | 0.961-2.075 | 0.079 |
| Deep Vein Thrombosis | 1.002 | 0.663-1.514 | 0.993 |
| Renal Insufficiency | 1.532 | 0.364-6.440 | 0.560 |
| Osteoporosis | 1.259 | 0.863-1.837 | 0.231 |
| Admission Hemoglobin | 0.990 | 0.981-0.999 | 0.026* |
| Non-early Surgery | 1.754 | 1.224-2.514 | 0.002* |

A P value<0.05 indicates statistical significance
